# The Human Papillomavirus E6 PDZ Binding Motif: From Life Cycle to Malignancy

**DOI:** 10.3390/v7072785

**Published:** 2015-07-02

**Authors:** Ketaki Ganti, Justyna Broniarczyk, Wiem Manoubi, Paola Massimi, Suruchi Mittal, David Pim, Anita Szalmas, Jayashree Thatte, Miranda Thomas, Vjekoslav Tomaić, Lawrence Banks

**Affiliations:** International Center for Genetic Engineering and Biotechnology, Area Science Park, Padriciano 99, Trieste 34149, Italy; E-Mails: ganti@icgeb.org (K.G.); justekbr@amu.edu.pl (J.B.); wiem.manoubi@yahoo.fr (W.M.); paola@icgeb.org (P.M.); mittal@icgeb.org (S.M.); pim@icgeb.org (D.P.); aszalmas@med.unideb.hu (A.S.); thatte@icgeb.org (J.T.); miranda@icgeb.org (M.T.); tomaic@icgeb.org (V.T.)

**Keywords:** HPV, E6, PDZ, 14-3-3, cell polarity

## Abstract

Cancer-causing HPV E6 oncoproteins are characterized by the presence of a PDZ binding motif (PBM) at their extreme carboxy terminus. It was long thought that this region of E6 had a sole function to confer interaction with a defined set of cellular substrates. However, more recent studies have shown that the E6 PBM has a complex pattern of regulation, whereby phosphorylation within the PBM can regulate interaction with two classes of cellular proteins: those containing PDZ domains and the members of the 14-3-3 family of proteins. In this review, we explore the roles that the PBM and its ligands play in the virus life cycle, and subsequently how these can inadvertently contribute towards the development of malignancy. We also explore how subtle alterations in cellular signal transduction pathways might result in aberrant E6 phosphorylation, which in turn might contribute towards disease progression.

## 1. Human Papillomaviruses and Cancer

Human Papillomaviruses (HPV) are the primary causal agents of cervical cancer and are linked to a growing number of other malignancies [1]. Of these cancers, the most important is cervical cancer of which there are over 500,000 cases worldwide annually [1,2]. The cancer causing HPV types are termed high risk types and the most frequently occurring HPV types in cervical cancer are HPV 16 and HPV 18 [3]. HPV is also the causative agent of cancers at a number of other anogenital sites [4] and in a rapidly increasing proportion of head and neck cancers [5]. The hallmark of HPV induced cancer is the persistent expression of the early proteins E6 and E7, which function as oncogenes throughout development of the malignancy [6,7]. Indeed, ablation of E6 and/or E7 expression in cervical tumor derived cell lines [8,9], primary cervical tumor cells [10] and also in tumors in transgenic mice [11] expressing the E7 oncoprotein, results in a cessation of transformed cell growth and tumor repression, manifested through senescence [12] or apoptosis [13]. Therefore, these viral oncoproteins are excellent targets for therapeutic intervention in HPV induced malignancy.

## 2. Differences between the High and Low Risk Viruses

There are close to 200 HPV types that have been described thus far, but among these only a few are associated with cancer. These high-risk viruses, which include the predominant cancer causing viruses HPV16 and HPV18, also includes 10 other types (31, 33, 35, 39, 45, 51, 52, 56, 58, and 59), which are also defined as cancer causing by the WHO [3]. The HPV types that do not cause malignancy are termed “Low risk” and include the HPV types 6 and 11, which are generally associated with benign epithelial condylomas. The question of why some viruses are cancer causing, while others are not has garnered a great deal of attention. Detailed analyses of various viral genomes demonstrate a high degree of conservation between the virus types, as well as conservation in many of the cellular targets of the viral oncoproteins. One of the most obvious differences between the E6 oncoproteins of the high and low risk HPV types is the presence of a C-terminal PDZ (PSD-95/DLG/ZO-1) binding motif (PBM) [14] in the E6 oncoproteins of high risk viruses that is absent in the low risk HPV types. This PBM, therefore, represents a molecular signature for the oncogenic potential of the high risk E6 proteins [15,16].

PDZ domains are stretches of 80–90 amino acids in length and were described from the three proteins that were first shown to harbor these domains *i.e.*, the Post Synaptic Density 95 (PSD95), the Discs Large (Dlg) and the Zona Occludens 1 (ZO-1) proteins. These domains are sites of protein-protein interactions with the ligands containing the so-called PDZ binding motifs (PBMs). Many of these proteins contain multiple copies of these PDZ domains and frequently also contain other protein-protein interaction motifs, thereby allowing these proteins to be involved in a plethora of different activities. Indeed, in the case of many PDZ domain containing proteins, they are considered to act as scaffolding proteins, facilitating the assembly of multi-protein complexes [17].

**Figure 1:**
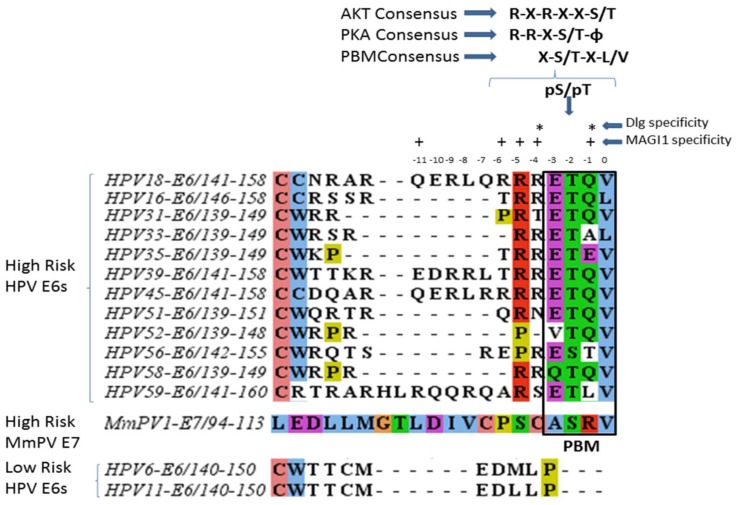
Papillomavirus oncoproteins show diversity in the PBMs and their kinase recognition sequences. The multiple sequence alignment of various HPV E6 proteins from different HPV types and the MmPV E7 using the Clustal X color scheme for the ClustalW sequence alignment program [28] show variation in the sequences of their C-terminus, which includes the PBM in the high risk HPV E6 proteins as well as the MmPV1 E7 protein, which is absent in the low risk HPV E6 types. The 4 boxed amino acids at the extreme C-terminus designate the canonical PBM. Whilst residues at p0 and -2 form the basis of PDZ recognition, the residues marked (*) indicate the amino acids that have been shown to be important for the specificity of E6 interaction with Dlg, while residues marked (+) designate the amino acids crucial for MAGI-1 specificity. The consensus sequences for AKT and PKA recognition of the E6 proteins is also shown.

The recognition of the PDZ domain is dependent upon the precise sequence of the PBM on the PDZ ligand. This PBM usually lies at the extreme C-terminus of the protein, but can also be found at internal sites [14]. There are three broad classes of PBM’s defined by their C-terminal consensus sequences: type I PBM (-X-S/T-X-Φ_COOH_), type II PBM (-X-Φ-X-Φ_COOH_), and type III PBM (-X-D/E-X-Φ_COOH_), where X is any residue and Φ is a hydrophobic residue [14,18,19]. However, there is a wide diversity in the types of PBM’s, and 16 distinct subtypes have been proposed [14,17]. Furthermore, PDZ domain-ligand specificity is not solely dependent upon the last four amino acids but involves at least the last seven carboxy terminal amino acids, with both canonical and non-canonical residues in the PBM contributing towards substrate specificity [20]. As can be seen from Figure 1, the high risk HPV E6 oncoproteins, while all possessing the canonical X-S/T-X-Φ_COOH_ consensus site, nonetheless display significant degrees of variation within this region. The first intimation that these changes might be biologically relevant came from early studies on the interaction of HPV 16 and 18 E6 with two potential PDZ domain substrates, the Discs Large (Dlg) and the Scribble (Scrib) proteins. In this case, a single Leucine/Valine substitution at the C terminal residue of the E6 PBM, was instrumental in determining substrate preference, with a C terminal Valine in 18E6 conferring preference for Dlg interactions, with a C terminal Leucine in 16E6 conferring preference for Scrib [21]. Indeed, both crystallographic and NMR studies performed under conditions where the HPV18 E6 PBM is in complex with two different PDZ domain-containing substrates, Dlg and the Membrane-associated guanylate kinase, WW and PDZ domain-containing protein 1 (MAGI-1), defined the importance of non-canonical residues within the PBM for contributing towards substrate specificity [22,23,24]. As can be seen from Figure 1, apart from the key residues at p0 and p-2 which define the PBM, residues at p-3, p-4, p-5 and p-6, and in the unique case of HPV-18 E6 at p-11, play essential roles in determining substrate specificity in the recognition of both Dlg and/or MAGI-1. Interestingly, most of these residues are non-canonical and lie outside the core PBM consensus sequence. As can be seen, there is also a great degree of variation in the sequence of these residues among the different high risk E6 oncoproteins. This highlights the fact that there is a considerable variability in the way these E6 proteins recognize diverse PDZ domain substrates.

High resolution NMR studies show that the upstream flanking regions of the PDZ1 domain of MAGI-1 increase the binding affinity to the PBM of E6 and also shows the PBM to have a disordered structure which becomes structured upon binding to the PDZ domain [22]. The number of critical contact sites is reflected directly in the strength of association between E6 and its different PDZ targets, with dissociation constants lying in the micromolar range with regard to MAGI-1, with a K_D_ value of about 3.0 μM for 16E6 and 1.5 μM for 18E6 [23], with again the difference in the C-terminal residue playing a significant role. Taken together, these studies indicate conservation of the PBM across high-risk E6 types, although the identity of preferred PDZ domain substrates of these different HPV E6 oncoproteins is likely to vary. As will be seen from the following discussion, this is indeed borne out by the fact that different E6 oncoproteins target a large number of different PDZ domain containing proteins, many of which are involved in the control of the same cell signaling pathways [25] (Table 1). Recent proteomic studies also have identified potential novel interacting partners of HPV E6 that play a role in pathways other than cell polarity [25,26,27], such as endosomal transport (Table 1).

**Table 1:** Known PDZ Domain containing targets of E6.

Protein Name	Function	Reference
DLG1	Polarity/tumour suppressor	[15,16,21,29,30,31,32,33,34]
SCRIB	Polarity/tumour suppressor	[21,35,36]
MAGI1/2/3	Polarity/tumour suppressor	[24,32,37,38]
PSD95	Signaling complex scaffold	[39]
TIP2/GIPC	TGF-β signaling	[40]
NHERF1	PI3K/AKT signaling	[41]
MUPP1	Signaling complex scaffold	[42]
PATJ	Tight junction assembly	[43,44]
PTPH1/PTPN3	Protein tyrosine phosphatase	[45,46]
PTPN13	Non-receptor phosphatase	[47]
PDZRN3/LNX3	RING-containing ubiquitin ligase	[48]
14-3-3	Signaling complex adapter	[49,50]
PAR3	Polarity/tumour suppressor	[51]
SNX27	Endosomal trafficking/signaling	[26,27]
ARHGEF12	RhoGEF	[26]
FRMPD2	Tight junction formation	[26]
LRRC7	Normal synaptic spine function	[26]

A critical aspect of HPV E6 is its multifunctionality. This protein has been reported to interact with at least 30 different cellular substrates [52], and these are involved in all aspects of E6 activity, from roles in the virus life cycle to the development of malignancy. For example, interaction with the cellular ubiquitin ligase, E6AP would appear central to many of E6 activities, by allowing E6 to direct its targets for proteasome mediated degradation [52]. However, this interaction also appears to play a central role in regulating E6 stability and turnover, presumably through holding the E6 structure in a stable conformation [53,54,55,56]. Another critical interaction involves the p53 tumor suppressor, and the ability of E6 to target and degrade p53 appears essential for multiple E6 functions [52]. However, it is also clear from the E6 structure that E6 interaction with E6AP and p53, as well as many of its other substrates, is very complex and often involves overlapping interaction sites [54,55,56,57]. This makes elucidating the role of these interactions in HPV E6 biological functions very complex, as mutations which abolish one set of interactions are likely to affect other substrates, thereby making interpretations as to which interactions are relevant for each activity very difficult. In contrast, the PBM is a highly defined region of the E6 oncoprotein and structural studies indicate a disordered structure, well away from E6’s other protein interaction sites [57]. This makes studies to understand the function of this region of E6 much easier as it allows specific mutations to be introduced in the PBM without affecting any of E6’s other activities [58,59]. This is exemplified most clearly in studies on the virus life cycle.

## 3. The E6 PBM and the Viral Life Cycle

**Figure 2:**
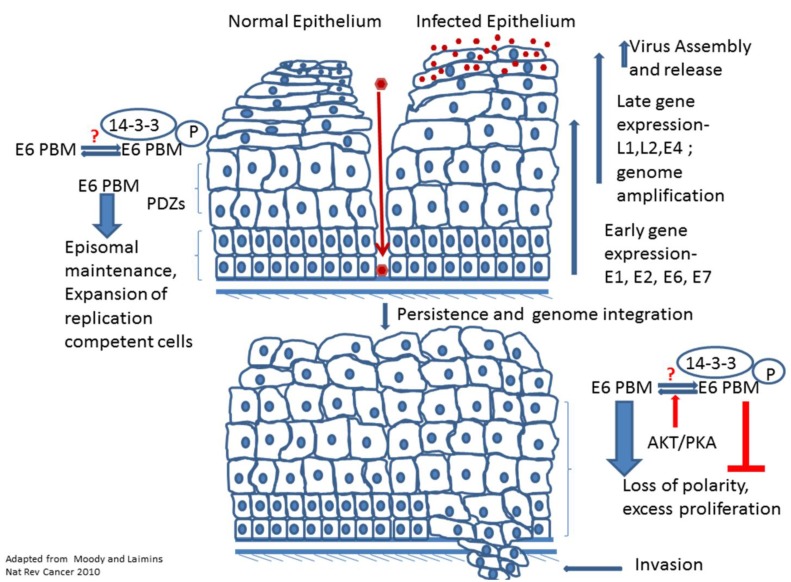
The role of the E6 PBM in the HPV life cycle and malignancy. The figure shows the productive life cycle of the virus after infection of the epithelium with coordinate expression of the different viral gene products during epithelial differentiation, ultimately resulting in the production of new infectious virus particles. The E6 PBM function, most likely through PDZ targeting, is required for expansion of replication competent cells and for maintenance of the viral episomal DNA. During differentiation and viral DNA amplification in the G2M like phase of the cell cycle, E6 will most likely be phosphorylated within the PBM, which could confer interaction with 14-3-3 proteins. Following a persistent infection of up to 20 years, the progression of HPV induced malignancy can occur. The role of the E6 PBM in this stage is unknown, but PDZ targeting might contribute to loss of cell polarity regulators and drive proliferation and invasion. Phosphorylation of the E6 PBM might be a means of negatively regulating this activity of E6.

The productive phase of the HPV life cycle is intimately linked with the differentiation program of the host epithelium [60]. The virus infects the basal cells of the epithelium via micro abrasions (see Figure 2), where multiple viral genome copies are maintained as persistent episomes. The division of these infected cells and their movement through the upper strata of the epithelium as they differentiate is coupled with the expression of different viral proteins including E6, and with the amplification of viral DNA [61]. Studies with HPV genomes lacking E6 show that these genomes are unable to establish themselves as episomes within the cell [62,63]. It is speculated that the roles of E6 in the viral life cycle range from the inhibition of apoptosis that is induced by the E7 oncoprotein to the facilitation of viral DNA amplification within the upper epithelial strata [64]. Studies in Human Foreskin Keratinocytes (HFKs) transfected with HPV 31 genomes have shown that the presence of the E6 and E7 oncoproteins is crucial for the stable replication of viral DNA, but not for transient replication [63]. The maintenance of viral copy number and proliferation, as well as episomal maintenance of viral DNA in undifferentiated cells, also requires the presence of the PBM, as demonstrated by the reduced replicative capacity and growth rates of HFKs stably maintaining PBM mutant genomes of HPV 31 [34].

HFKs containing HPV 18 genomes also displayed similar phenotypes, with the cells expressing E6 ΔPBM containing genomes proliferating somewhat more slowly than the cells containing wild type HPV 18 genomes. Furthermore, in organotypic cultures, the E6 ΔPBM genomes exhibited defects in the levels to which the viral genomes could be amplified and a corresponding decrease in the number of suprabasal cells that were capable of replicating DNA. Most interestingly, extended periods of passaging resulted in a loss of E6 ΔPBM genomes, indicating that the E6 PBM plays an essential role in episomal maintenance and in creating an environment favorable for viral DNA replication [65].

Normal Immortal Keratinocytes (NIKS) containing HPV 16 genomes with E6 ΔPBM mutations also exhibited significant defects in the viral life cycle. However, in this particular instance, this was correlated with decreased levels of expression of the HPV 16 E6 protein [66]. This suggested that at least one of the E6’s PDZ target proteins might play a role in regulating E6 stability or levels of expression. In support of this, loss of Scrib in HeLa cells was found to greatly reduce the levels of HPV 18E6 protein. However, the molecular basis for this effect remains to be determined [66]. It was also observed that the HPV 16 E6 ΔPBM genomes failed to be maintained as episomes in NIKS cells, compared with wild type genomes.

Recent studies in NIKS containing either wild type HPV16 genomes or genomes with mutant forms of E6 that were either prematurely terminated to ablate all forms of E6 or were lacking just the last two amino acids of the PBM, thus making them unable to interact with PDZ proteins, show that, although the PBM defective mutant is not maintained stably as episomes in these cells through extended passages as described previously [66], this phenotype can be reversed by the repression of p53. The repression of p53, either through shRNA or through the expression of a dominant negative form of the protein, stabilizes the PBM defective 16E6 mutant genome in these NIKS, and the genome is now capable of being maintained as a stable episome [67]. These results are particularly intriguing since the PBM deletion mutants of E6 target p53 as efficiently as wild type E6. This indicates that at least one aspect of E6 PDZ targeting links directly to pathways that are controlled by p53 [67,68]. It is also possible that the PDZ-PBM interaction of wild type E6 contributes towards the abrogation of a p53 function that is independent of the degradation of p53, and this functional ablation is necessary for viral genome maintenance. Clearly, elucidating the molecular basis for the link between PDZ targeting and p53 is a particularly exciting avenue of future research.

Taken together these studies indicate that the E6 PBM plays an essential role in the virus life cycle, being required for expansion of the population of replication-competent cells and thereby increasing the number of cells in which viral genome amplification can occur. A recent intriguing study demonstrated that evolution of the E6 PBM most likely preceded acquisition of an oncogenic phenotype, suggesting the possibility that the PBM may also play an important role in specific tissue tropism or niche selection [69], something that seems to be supported by studies described below in which the biological effects of the E6 PBM varies, depending upon the tissue or origin of cells.

## 4. The Role of the PBM in Transformation and Cancer

The ability of the high risk HPV E6 oncoproteins to effectively recognize and bind to cellular PDZ domain containing targets has been shown to be important for the transformation potential of E6, both *in vitro* [16,70] and *in vivo* [58], although differences are observed depending on cell type and the anatomical site. Studies in immortalized human keratinocytes that the HPV 18E6 transfected keratinocytes show an exaggerated epithelial to mesenchymal (EMT) phenotype and changes in actin cytoskeletal organization that are significantly reduced in the cells that contain HPV 18E6 PBM mutant cDNA. Furthermore, the wild type E6 expressing cells also show aberrant adherens junction and desmosome formation not observed in the E6 PBM mutant containing cells, implying that the E6 PBM contributes towards the development of transformed characteristics in primary keratinocytes [70]. Likewise, the E6 PBM has been shown to contribute towards anchorage-independent growth in murine and human tonsillar keratinocytes, as well as contributing to their immortalization [47]. The E6 PBM is also necessary for the inhibition of apoptosis in human airway epithelial cells [71]. In the case of rodent cells, the E6 PBM appears to contribute towards the ability of E6 to promote transformation, although this is not observed in mammary epithelial cell or foreskin keratinocyte immortalization assays [16,34,65,72,73]. This indicates that the E6 PBM itself plays a limited role in immortalization *per se*, most likely functioning in the later stages of malignant transformation; however, there are also clear elements of context dependence, where the E6 PBM has a more active role in certain cell types and/or contexts than in others.

Transgenic mice have been used extensively to dissect the role of E6 and E7 in the development of malignancy. This has been based on the expression of the viral oncogenes from a keratin 14 (K14) promoter, which ensures expression of the viral proteins in the correct cell type *i.e.*, basal cells of the cutaneous and squamous epithelia. Mice expressing HPV 16 E6 in the epidermis develop epithelial hyperplasia, which progresses to squamous carcinoma. In contrast, transgenic mice that express an E6 ΔPBM mutant, fail to develop hyperplasia, although they show a radiation response similar to the mice expressing wild type 16E6, as demonstrated by the lack of p53 or p21 induction upon irradiation. This indicates that both the wild type and the E6 ΔPBM mutant are capable of targeting and degrading p53, but that the ability to cause hyperplasia and promote progression towards a transformed phenotype is dependent on the presence of an intact and functional PBM [58]. In the cervix, HPV16 E6 and E7 promote the development of tumors in the presence of chronic estrogen treatment [74,75]. Additionally important to note is that although E6 expressing mice develop tumors in the presence of estrogen, E7 is more efficient in initiating tumor formation in a similar setting, indicating that E6 most likely plays a greater role later in tumor progression [74]. In addition, mice that express wild type 16E6 alone develop cervical tumors at a greater frequency if estrogen treatment is extended to 9 months, whilst the E6 ΔPBM mice have fewer and smaller tumors [59]. There is also a significant reduction in the tumor size and multiplicity of tumors in mice expressing the PBM deletion mutant in co-operation with wild type E7 when compared with mice that express wild type E6 and E7 [59].

Studies with transgenic mouse models of head and neck cancer, as well as models of anal cancer, differ from those of the skin and cervix with respect to the potential role of the E6 PBM in the development of these cancers [76,77]. In the case of head and neck cancer, there is a co-operation between the E6 and E7 oncoproteins in the induction of squamous cell carcinoma, with E7 being the more potent oncogene in the presence of the chemical carcinogen 4-nitroquinoline-1-oxide (4-NQO) [76,78]. The studies with mice that express the E6 ΔPBM, showed that the ability of E6 to co-operate with E7 to cause head and neck squamous cell carcinoma (HNSCC) does not require an intact PBM function [76]. These results indicate that although E6 and E7 synergize to induce HNSCC in transgenic mice, this most likely involves a mechanism that is independent of the E6 PDZ binding potential. Similar phenotypes have been observed in mouse models of anal carcinoma, where HPV16 E7 is more effective in inducing proliferation in the anal epithelium compared with mice expressing 16 E6 alone when treated with the chemical carcinogen DMBA. Additionally important to note is that the co-expression of E6 and E7 in these mice does not seem to increase tumor induction rate or size compared with mice expressing E7 alone [77]. These observations again implicate E7 as the more potent oncogene in the development of anal cancer similar to HNSCC, although further studies with mutant E6 mice and a reduction of the treatment time with DMBA in the case of anal cancer may reveal potential roles of E6 in anal carcinogenesis.

These studies demonstrate a potent contribution of the E6 oncoprotein and the E6 PBM towards the development of certain malignancies in transgenic mice. However, the precise contribution of the PBM varies depending upon the anatomical site, again indicating a degree of context dependence in which the E6 PBM functions. This context dependence is a recurring theme in cell polarity signaling pathways; whose control is largely governed by PDZ domain containing proteins.

## 5. E6, Cell Adhesion and Polarity

Epithelial tissues have a characteristic polarized cellular architecture and specialized cell-cell junctions, including desmosomes, tight and adherens junctions. There are a number of signaling and polarity complexes that are involved in the recruitment of proteins to the junctions and in the establishment of cell polarity [79]. Cell polarity plays a crucial role in the organization of signaling pathways, which allow the interpretation of signals from the surrounding microenvironment, thus enabling the control of proliferation, metabolism, apoptosis, differentiation and motility [80]. Cell polarity is maintained by an intricate interplay between conserved groups of proteins that form distinct complexes:The Crumbs, Par and Scribble complexes. Apical polarity is controlled by the Crumbs complex, made up of Crumbs3, Pals1 and PatJ [81,82]. The Par complex consists of Par3, Cdc42, Par6 and atypical protein kinase C (aPKC), which is a dynamic complex and which also interacts with the Crumbs complex [80]. The Scribble complex consists of the scaffolding proteins Scribble, Dlg and Hugl, which maintain basolateral polarity. Of these cell polarity proteins, PatJ, Par3, Scrib and Dlg all possess PDZ domains and, hence, are potential targets of E6 [83]. It is important to remember that the control of cell polarity hinges on the correct spatio-temporal regulation of the expression levels of the cell polarity regulators. Alterations in expression levels or mislocalization of any of these components perturbs the function of the complex as a whole and leads to aberrant signaling that may be a driver of neoplastic transformation [84].

**Figure 3:**
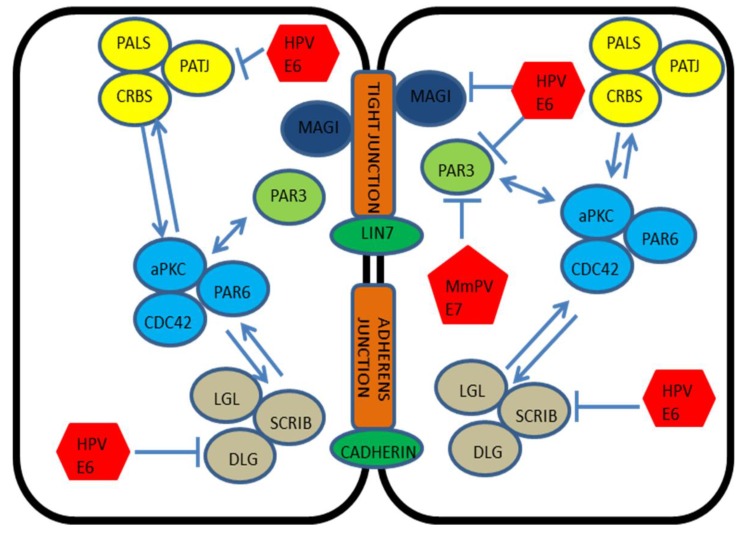
Papillomavirus oncoprotein targeting of cell polarity proteins. The cartoon depicts the various proteins comprising the three major complexes that regulate cell polarity: apical is defined by the Crumbs (CRBS) complex, subapical by the Par complex and basolateral by the Scrib complex. These complexes interact through a series of mutually antagonistic interactions ensuring correct spatial distribution and levels of expression of the individual components. Note the propensity of HPV E6 and MmPV E7 to target diverse components of this cell polarity control network, thereby perturbing their levels of expression or subcellular distribution.

One of the primary characteristics of the transition of a benign neoplasm to a malignant phenotype is a major disorganization of cellular architecture, which includes the loss of cell-cell contact and polarity, either through the degradation or mislocalization of the components that regulate these processes. The impact of the loss of polarity can be profound- the perturbation of the trafficking of proteins to the apical or basolateral regions may cause aberrant signaling due to mislocalization of receptors, or a redistribution of cell adhesion molecules that can promote cellular transformation in EMT. Altered polarity can also lead to an inappropriate distribution of degradative enzymes such as matrix metalloproteinases at the cell surface, thus promoting cell invasion and transformation, as well as affecting migration and cytoskeletal organization [85]. Many of the key regulators of cell polarity and adhesion are PDZ domain containing proteins, a number of which have been reported to be targets of high risk HPV E6 oncoproteins, albeit with varying affinities (See Figure 3), and these include the core components of the cell polarity control machinery; the Scribble-Dlg [21,33,36], the Par-aPKC [51,86], and the Crumbs complexes [43,44]. Furthermore, a number of cell junction proteins are targeted by E6 and these include the MAGI group of proteins [37,38], all of which are crucial for maintaining junctional stability and integrity.

The Scribble-Dlg axis appears to be one of the *bona fide* targets of the high risk E6 oncoproteins, with numerous studies showing that both proteins can be bound and degraded by E6 *in vitro* and *in vivo* [33,36,87]. It has also been observed that overexpression of Scrib inhibits the transformation of rodent epithelial cells by high risk E6 and E7 proteins, indicating a potential role for Scribble as a tumor suppressor [21,36,58]. Indeed, deregulation of Scribble function is seen in a wide range of epithelial cancers- including colorectal [88] , breast [89], prostate [90], and endometrial cancers [91]. In the case of cervical cancer, the expression patterns of Scrib and Dlg are severely perturbed during tumor development [92,93,94]. A general trend during the development of cervical cancer is the unusual cytoplasmic distribution of Dlg in cervical intraepithelial precursor lesions, as opposed to the cell-cell contact localization seen in normal tissue. In contrast, a loss of Dlg is observed only in late stage invasive cervical cancer [92]. This trend is also observed in the case of Scribble, where a redistribution of Scribble is observed from sites of cell contact in normal squamous cells to the cytoplasm in early dysplasia, followed by a steady reduction in protein levels as the tumor progresses [95]. Whether these perturbations to Dlg and Scrib expression during the progression of cervical cancer are due to the effects of E6 is still an open question and subject of intense research.

It is important to note that E6 does not induce the degradation of the entire pool of Scribble and Dlg, but instead only targets a specific subset of the proteins. In the case of Dlg and HPV 18E6, only phosphorylated and nuclear fractions of Dlg appear to be targeted for proteasome mediated degradation. Paradoxically, both Dlg and Scribble show pro-oncogenic activity in certain contexts [96,97]. For example, the adenovirus E4 protein triggers Dlg translocation to the plasma membrane of epithelial cells where it goes on to activate PI3K, with the assembly of a ternary complex made up of Dlg1, PI3K, E4 and Akt [98,99]. This membrane associated PI3K is constitutively active and mediates Akt signaling which is associated with tumorigenesis. Scrib has also been found to be overexpressed and mislocalized in breast cancer and has been linked to a gain of pro-oncogenic activity [100]. It is also interesting to hypothesize that such pro-oncogenic functions of PDZ proteins can be manipulated by the HPV E6 protein to create a favorable environment for cellular proliferation.

Indeed, recent studies showed that E6 stimulates RhoG activity by a mechanism that is dependent upon its interaction with Dlg and the RhoG guanine nucleotide exchange factor SGEF. Thus, in HPV positive cells, there are high levels of RhoG activity, resulting in increased invasive potential, which is directly dependent upon continued expression of E6, Dlg and SGEF [101]. These findings demonstrate a hitherto unknown pro-oncogenic function of Dlg in HPV transformed cells. It is thus tempting to speculate that the mislocalization or overexpression of Dlg or Scrib may induce a switch from tumor suppressor to a pro-oncogenic function, possibly due to the alteration in the pool of their interacting partners, thus modulating their function, especially in the case of intermediate grade tumors, thus increasing their potential for progression to invasive cancers [102].

The MAGI family of proteins has been shown to be one of the most strongly bound and most susceptible groups of proteins to be targeted by the high risk E6 proteins [22,37,38,103,104]. MAGI-1 in particular is a major degradation target of both HPV 16E6 and HPV 18E6 [87]. As in the case of Dlg, only certain pools of the protein are targeted, with the membrane and nuclear pools of MAGI-1 being susceptible to proteasome-mediated degradation by both HPV 16E6 and HPV 18E6. The loss of TJ integrity is a direct consequence of the loss of MAGI-1 in cervical cancer cell lines HeLa and CaSKi, which is reinstated upon E6 ablation and the re-emergence of MAGI-1 expression in these cells [87]. It is also interesting to note that the reintroduction of a mutant MAGI-1 that is resistant to E6 induced degradation in HPV positive cells leads to the accumulation of ZO-1 and Par3 at cell contact sites, as well as a significant reduction in cell proliferation and an increase in the number of apoptotic cells [105]. This finding sheds light upon the pathological consequence of the loss of MAGI-1 in HPV positive cells, which includes loss of tight junction stability, an increase in proliferation and a suppression of apoptosis, all of which can be expected to enhance the progression of hyperplastic lesions into metastatic cancer. Interestingly, from a life cycle point of view, this dissociation of the control of cell proliferation by contact inhibition might be means by which E6 targeting of these junctional complexes can be expected to induce proliferation of the suprabasal epithelial cells during the virus life cycle (See Figure 2).

The only other Papillomavirus known to cause cervical cancer in its natural host is the *Macaca mulatta* Papillomavirus type 1 (MmPV1). This virus is very similar to HPV16, is sexually transmitted and causes cervical cancer in rhesus macaques [106,107,108]. However, unlike the high risk HPVs, the E6 protein of MmPV1 does not contain a C-terminal PBM. Instead, a class I PBM (ASRV) is present on the C-terminus of the E7 protein of this virus. As can be seen from Figure 1, the sequence of the E7 PBM is quite distinct from that found in the high risk HPV E6 proteins [86], however it nevertheless fits perfectly within the consensus sequence. Not surprisingly, such a difference in the PBM is reflected in differences in the PDZ proteins bound by MmPV1 E7, with only very weak levels of interaction seen with Scribble and Dlg. Indeed, the preferred PDZ substrate of the MmPV1 E7 protein is Par3, which, as noted above, is a critical component in the cell polarity control pathway. Par3 defines the sub-apical region of the cell and maintains apico-basal polarity in conjunction with the Crumbs and Scribble complexes and therefore belongs to the same polarity control pathway as Scrib and Dlg [86,109,110]. Whilst there is no information available on the status of Par3 in Rhesus macaque cervical cancers, it is clear that MmPV1 E7 perturbs the pattern of Par3 expression in cultured cells, and can therefore be expected to perturb the correct functioning of the cell polarity network. This interaction of MmPV1 E7 with Par3 is also biologically significant, as Par3 binding-defective mutants of MmPV1 E7 lose their ability to transform primary rodent cells [86]. It thus appears that the high risk HPVs and MmPV1 target the same pathway of polarity control using a similar mechanism of PDZ recognition, although they target different components of the pathway through the action of different viral oncoproteins, indicating a high degree of evolutionary conservation across these different cancer causing Papillomaviruses.

## 6. The Multifunctionality of the E6 Oncoprotein

As noted above, the E6 oncoproteins are multifunctional with many different interacting partners required for their multiplicity of function, both during the changing environment of the virus life cycle and during cancer development. Not surprisingly, the E6 PBM is equally multifunctional. Embedded within the PBM of all of the high risk HPV E6 oncoproteins is a potential phospho acceptor site (see Figure 1). It was first shown for HPV 18E6 that Protein Kinase A (PKA) could very efficiently phosphorylate the Threonine residue at position 156 within the PBM, and this in turn resulted in a dramatic inhibition of the ability of E6 to interact with its PDZ substrate Dlg [111]. This is in accordance with observations that phosphorylation of the PBM generally abrogates PDZ-PBM interactions and this is because the phospho moiety cannot be accommodated in the PDZ binding pocket [112]. Not surprisingly, this phospho-dependent inhibition of PDZ recognition is also true for other HPV E6 oncoproteins and other PDZ domain containing substrates [49]. What is more surprising is that the regulation of the different HPV E6 PBMs is not controlled by the same kinase. For example, HPV 18E6 is only phosphorylated by PKA, whilst HPV 16E6 can be phosphorylated by PKA or AKT [49]. A similar situation also holds true for some HPV E6 proteins from other virus types. This raises the surprising possibility that there are significant differences in how the different HPV E6 oncoproteins are regulated. For example, AKT levels are high in proliferating cells [113], whilst PKA levels are high during differentiation [65,70], and this suggests subtle differences in the mechanisms by which the interaction of the high risk HPV E6 oncoproteins with the different PDZ substrates are modulated.

So, does the phosphorylation of the PBM simply act to negatively regulate PDZ recognition or can it confer an additional function upon the E6 protein? Indeed, recent studies have shown that phosphorylation of the E6 PBM confers a strong direct interaction with members of the 14-3-3 family of proteins [49]. 14-3-3 proteins are a group of highly conserved acidic proteins. There are seven known isoforms of 14-3-3 present in mammals that are encoded by seven distinct genes. 14-3-3 proteins bind to a large repertoire of proteins mainly in a phospho-specific manner [114,115,116,117]. 14-3-3 proteins function as adapter proteins and interact with a plethora of cellular proteins involved in a wide variety of processes, including signal transduction, apoptosis, metabolic control, cytoskeletal maintenance, tumor suppression, and transcription [118]. The interaction of E6 with 14-3-3 in a phospho-specific manner thus raises intriguing questions as to its role in the modulation of 14-3-3 function or vice versa. The phosphorylation of E6 confers preferred association with 14-3-3 zeta, which in turn seems to be important for maintaining E6 stability in HeLa cells [49].

It therefore seems likely that the E6 PBM function will be differentially regulated through the progression of the viral life cycle, both in the context of recognition of different PDZ containing substrates as well as its interaction with phosphorylation-dependent cellular proteins, such as 14-3-3. Indeed, mutation of the PKA consensus recognition site in HPV 18E6, in the context of the whole genome in organotypic cultures, leads to a more hyperplastic and stratified phenotype, most likely as a result of conferring constitutive interaction with PDZ domain containing substrates [65]. So, what do these observations mean in the context of the fine regulation of E6 PBM function during the course of the viral life cycle and in the development of malignancy? Certainly it is plausible that changes in cellular signal transduction pathways might be reflected in altered patterns of phosphorylation of E6. This in turn could promote or restrict malignant progression, depending upon the specific situation. Since 14-3-3 proteins are heavily involved in the regulation of the cell cycle, it also raises the possibility that the interaction of E6 with 14-3-3 may be modulating its function so as to maintain an environment favorable for viral genome amplification, as for example in the G2/M phase of the cell cycle even in the absence of appropriate signals. It is also tempting to speculate that this intricate phospho-regulation may have arisen as a way of allowing compartmentalization of E6 function during the various stages of the viral life cycle. In this way, phosphorylated E6 will be sequestered by the 14-3-3 proteins and will thus be unable to target the PDZ proteins that are crucial for maintaining structural integrity of the infected cell and appear to be required for promoting cellular proliferation. It is also plausible that the aberrant regulation of E6 phosphorylation may be a prognostic marker for the predisposition of benign lesions to progress into invasive cancer. Whether this is due to a lack or gain of phosphorylation is obviously an aspect requiring further investigation. Future studies involving the dissection of the role of phospho-E6 and its possible effects on 14-3-3 activity, both during the virus life cycle and during the progression to malignancy, should yield fascinating insights into the function of this highly dynamic and multifunctional region of the E6 oncoprotein.
